# Clinical Characteristics of Minor Hallucinations in Chinese Parkinson's Disease Patients

**DOI:** 10.3389/fnagi.2021.723405

**Published:** 2022-01-20

**Authors:** Yu Zhang, Guo yong Zhang, Xiao bo Zhu, Zi en Zhang, Jing Gan, Zhen guo Liu

**Affiliations:** Department of Neurology, Xinhua Hospital Affiliated to Shanghai Jiao Tong University School of Medicine, Shanghai, China

**Keywords:** Parkinson's disease, minor hallucinations, psychosis symptoms, non-motor symptoms, China

## Abstract

**Background:**

Psychotic symptoms are common in Parkinson's disease (PD). However, the clinical characteristics of PD psychosis (PDP) have been rarely reported in Chinese PD patients. We aimed to categorize PDP in a PD cohort and its relationship to other clinical characteristics.

**Methods:**

A total of 149 Chinese PD patients were consecutively enrolled, and idiopathic PD patients were recruited in the study. The symptoms of PDP were assessed with the enhanced Scale for the Assessment of Positive Symptoms in PD. Then, the patients were classified into a PD-control group, isolated minor hallucination (MH) group, and complex MH group, and clinical and demographic data of different groups were compared.

**Results:**

Parkinson's disease psychosis was present in 40.3% (60/149) of our patients. The most common PDPs were MHs, present in 32.9% (49 of 149) of the cohort. Compared to patients without MHs, patients with MHs were older, had a longer disease duration, a higher levodopa equivalent daily dose, more severe motor symptoms, dyskinesia, a higher rate of rapid eye movement sleep behavior disorders, frontal lobe function impairments, and a higher percentage of cognitive impairment. Logistic regression analysis showed that advanced Hoehn-Yahr stage [odds ratio (OR): 2.697, *p* = 0.007)] and frontal lobe function impairment (OR: 0.684, *p* = 0.003) were independent risk factors for MHs.

**Conclusion:**

MHs were frequent non-motor symptoms in PD patients. It was associated with increased motor and non-motor symptom burdens and reduced quality of life. MHs have been called “minor,” but they have major clinical and prognostic implications and need early screening.

## Introduction

Parkinson's disease (PD) psychosis (PDP) is one of the debilitating non-motor symptoms (NMSs) of PD. There is a spectrum of PDP symptoms, including minor hallucinations (MHs), hallucinations of various sensory modalities (visual, auditory, olfactory, gustatory, and tactile hallucinations), and delusions (Frei and Truong, 2017). Most studies of PDP have focused on well-structured hallucinations (Zhu et al., 2017; Eversfield and Orton, 2019), which could be linked to a higher mortality (Forsaa et al., 2010; Zhu et al., 2017; Clegg et al., 2018; Eversfield and Orton, 2019; Lenka et al., 2019). However, there is a growing concern of MHs of PD patients by clinicians, which was first mentioned by Fenelon et al. (2000).

Minor hallucinations (MHs) include misperceptions as a sense of presence, passage, and illusions. It is considered the mildest and earliest form of PDP. PD patients could have one or more minor hallucinations (isolated MHs) or have minor hallucinations along with other sensory modality hallucinations (complex MHs) (Fenelon et al., 2000; Frei and Truong, 2017; Kulick et al., 2018). Clinically, the subtle symptoms of MHs have often been ignored. However, a previous study reported that PD patients with MHs more closely resembled patients with hallucinations or delusions (Kulick et al., 2018). This could be explained by a neuroimaging study, which showed similar structural and functional changes in patients with MHs or well-structured hallucinations (Mack et al., 2012; Pagonabarraga et al., 2016; Ffytche et al., 2017; Bejr-Kasem et al., 2019). However, the prevalence and the clinical profile of MHs have not been clearly elaborated. Therefore, we aimed to categorize PDPs in a PD cohort and their relationships to other clinical characteristics.

## Methods

### Patients

From January 2019 to December 2019, 149 patients with PD were consecutively recruited from the Department of Neurology, XinHua Hospital Affiliated with JiaoTong University, School of Medicine, Shanghai, China, after obtaining their written informed consent. These PD patients all met the criteria of the Movement Disorder Society PD Criteria (Postuma et al., 2015), and had enough audiovisual functions to complete motor and non-motor symptom evaluation tests. Participants were excluded if they had possible dementia with Lewy bodies (DLB) according to the 2005 DLB diagnostic criteria (McKeith et al., 2005). We excluded patients with secondary Parkinsonism, patients after deep brain stimulation, patients with a history of other neurological, psychiatric, or severe ocular diseases, and patients with MRI evidence of focal brain lesions. No patient was taking antipsychotics. The PD patients with Minimum Mental State Examination (MMSE) <24 were also excluded. This study was approved by the Ethics Committee of XinHua Hospital Affiliated to Shanghai Jiao Tong University School of Medicine.

### Clinical Assessment

A structured interview for clinical and demographic variables was performed and medical information was collected. The data of patients included sex, age, age of motor onset, predominant symptoms at onset, disease duration, history of smoking and alcohol consumption, and dopaminergic administration and related complications. Total levodopa equivalent daily dose (LEDD) was calculated according to the previously suggested conversion formula (Fenelon et al., 2000). The motor symptoms were assessed with the Unified Parkinson's Disease Rating Scale part III (UPDRS-III) and Hoehn-Yahr (H-Y) stage. The NMSs were measured with the following scales: MMSE, the Parkinson's Disease Sleep Scale (PDSS), REM Sleep Behavior Disorder Questionnaire Hong Kong, Hamilton Depression Scale (HAMD), and the Frontal Assessment Battery (FAB). The quality of life was assessed using the Parkinson's Disease Questionaire-39 score (PDQ-39).

Psychotic phenomena that patients experienced at least weekly during the last month were assessed in an interview of patients and their caregivers by two experienced neurologists. The psychiatric symptoms were assessed with the enhanced Scale for the Assessment of Positive Symptoms in PD (eSAPS-PD), which was more sensitive for PDP than other common clinical measures (Kulick et al., 2018). The eSAPS-PD was administered for approximately 2 min in patients who reported no psychotic symptoms, and over 10 min in those with numerous or complex psychotic symptoms. Patients were classified as (1) the isolated MHs group, if patients had ≥ 1 MHs but no well-structured hallucinations and delusions (eSAPS-PD scores ≥ 1 on question 1, with score = 0 on questions 2–13); (2) the complex MHs group, if patients had MHs concomitant well-structured hallucinations or delusions (eSAPS-PD scores ≥ 1 on question 1 and scores ≥ 1 on questions 2–13); and (3) the PD-control group, if patients had no psychotic symptoms (eSAPS-PD score = 0).

### Statistical Analysis

The data are expressed as the mean ± SD. The categorical statistics were recorded as count and percentage data. Descriptive statistics underwent a normality test. ANOVA with *post-hoc* comparisons was performed for group differences across MH subgroups and PD-control groups. The chi-squared test or *t*-test was used to analyze group differences between patients with and without MHs. A binary logistic regression analysis based on a forward stepwise method was conducted to determine the most significant variables, which were independently correlated with MHs in PD patients, especially with complex MHs. The level of significance was set at *p* < 0.05. The odds ratio (OR) was presented with 95% CIs. The multicollinearity was absent for the model. To determine the predictive value of combined multivariate observations in the occurrence of complex MHs in PD patients, the receiver operating characteristic (ROC) curve was used and the area under the curve (AUC) calculated, and the 95% CI. Youden's index was used to determine the best sensitivity and specificity values. The predictive value of combined multivariate observations in the occurrence of MHs in PD patients was also calculated. The data were analyzed by SPSS statistical software for Windows, version 22.0 (SPSS, Chicago, IL, USA).

## Results

### Demographic Characteristics in PDP

**Figure 1:**
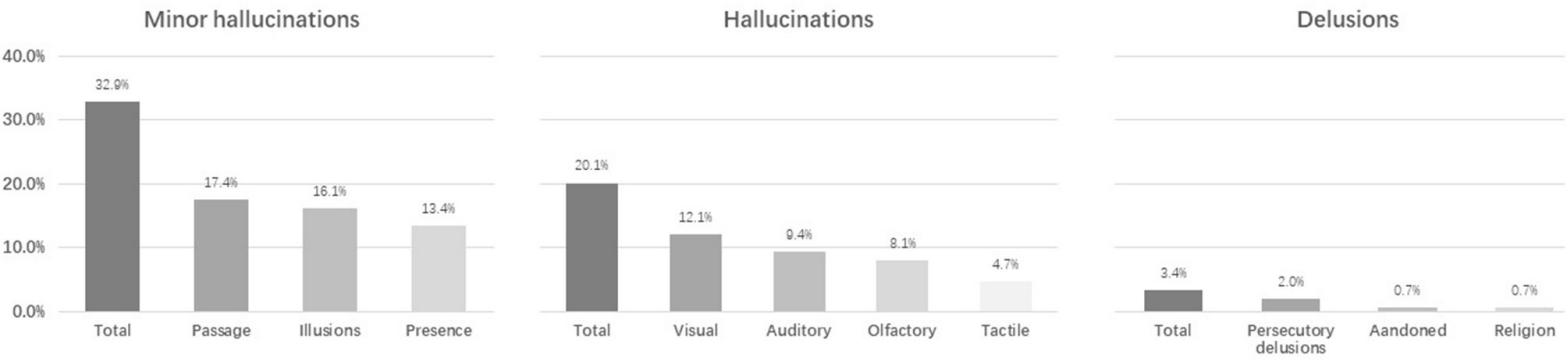
The frequency of various types of psychotic symptoms in the Parkinson's disease cohort.

A total of 149 consecutive patients with non-demented PD were included in our study. The mean age at the study was 68.41 ± 7.46 years, and the mean disease duration was 7.62 ± 4.27 years. The median H-Y stage was 2.5. We found that 60/149 (40.3%) had psychotic symptoms. A total of 49 of 149 (32.9%) PD patients had MHs, 30/149 (20.1%) had well-structured hallucinations, and 5/149 (3.4%) had delusions. The frequencies of various types of psychotic symptoms are shown in Figure 1.

Of the 49 PD patients with MHs, the most common MHs were passage hallucination. Twenty-six patients reported this type (26/49, 53.1%). They described them as a vision of a shadow, a person, or animals (running cats, rats, or dogs) passing sideways in the periphery of the visual field, and moving forward from behind the shoulder. Twenty-four patients reported illusions (49.0%). They were described as undefined images emerging from a patterned sofa or wallpaper, involving the movement of curtains. Twenty patients reported hallucinations (40.8%). They were described as the feeling of a known person behind the shoulder. In addition, 25 of 49 patients (51.0%) reported isolated MHs (occurring in 16.8% of the total patients). Both passage hallucinations (12 patients) and visual illusions (11 patients) were more frequent than presence hallucinations (seven patients). Of these 25 patients, five patients had more than one type of MH, and all of them had presence hallucinations, while 24 patients (24/49, 49.0%) had complex MHs (occurring in 16.1% of the total patients). Of these, 14 patients (14/24, 58.33%) had passage hallucinations, 13 patients had presence hallucinations, and 13 patients had illusions. Six patients had two types of MHs, and five patients had three types of MHs. Most patients had complex MHs (15/24, 62.5%) with concomitant visual hallucinations. Eleven of them (11/24, 45.8%) were concomitant auditory hallucinations, followed by concomitant olfactory (7/24, 29.2%) and tactile (6/24, 25.0%) hallucinations. Eleven of these patients with complex MHs (11/24, 45.83%) exhibited the presence of both hallucinations and other types of MHs.

The distribution of MHs was different in different H-Y stages. In patients with H-Y stages 1–2.5, 24.1% had MHs; this percentage was significantly lower than that at H-Y stages 3–5 (59.5%, χ^2^ = 15.749, *p* < 0.001). Similarly, 10.7% of patients in H-Y stages 1–2.5 had MHs concomitant with other hallucinations, which was significantly lower than that in H-Y stages 3–5 (32.4%, χ^2^ = 9.708, *p* = 0.002). However, no difference between H-Y stages 1–2.5 and stages 3–5 was found in the isolated MH group (13.4 vs. 27%, χ^2^ = 3.703, *p* = 0.054).

### Differences in PD Patients With and Without MHs

According to the scale of the eSAPS-PD, the patients were classified in the PD-control group, isolated MH group, and complex MH group (detailed in Methods). We compared the demographic and motor and non-motor characteristics of these three groups (Table 1).

**Table 1:** Characteristics of Chinese Parkinson's disease patients with and without minor hallucinations.

**Variables**	**PD-control group**	**Isolated MHs group**	**Complex MHs group**	***F*/χ^2^-**	***P***	**P1**	**P2**	**P3**
	**(*n* = 89)**	**(*n* = 25)**	**(*n* = 24)**	**Value**				
**Demographics**
Female sex, *n* (%)^a^	45 (50.6%)	9 (36.0%)	12 (50.0%)	1.714	0.425	–	–	–
Age (years)^b^	67.13 ± 7.54	69.32 ± 8.45	71.42 ± 7.19	3.469	0.034	0.194	0.013	0.323
Onset age (years)^b^	60.31 ± 7.92	60.52 ± 8.45	62.88 ± 8.62	0.954	0.388	–	–	–
Disease duration (years)^b^	6.84 ± 3.60	8.80 ± 4.94	8.54 ± 4.85	3.177	0.045	0.037	0.074	0.826
LEED (mg/day)^b^	451.28 ± 267.72	465.25 ± 234.95	654.69 ± 337.35	5.266	0.006	0.823	0.002	0.017
DA use, *n* (%)^a^	66 (74.2%)	23 (92%)	17 (70.8%)	4.072	>0.05	–	–	–
**Motor symptoms**
UPDRS-III scores^b^	18.81 ± 8.71	23.80 ± 13.25	29.33 ± 11.32	10.975	<0.001	0.031	<0.001	0.058
H-Y stage^b^	2.11 ± 0.55	2.52 ± 0.67	2.73 ± 0.66	12.714	<0.001	0.003	<0.001	0.219
Dyskinesia, *n* (%)^a^	6 (6.74%)	4 (16.0%)	5 (20.8%)	4.704	0.095	–	–	–
Wearing-Off, *n* (%)^a^	54 (60.67%)	11 (44.0%)	18(75.0%)	4.938	0.085	–	–	–
**Non-Motor symptoms**
FAB scores^b^	17.22 ± 1.33	15.84 ± 2.41	14.75 ± 3.42	15.399	<0.001	0.004	<0.001	0.065
MMSE scores^b^	27.78 ± 2.20	26.32 ± 3.31	26.08 ± 2.92	6.004	0.003	0.013	0.005	0.747
PDSS scores^b^	120.13 ± 17.15	115.56 ± 16.49	104.58 ± 23.31	6.924	0.001	0.270	<0.001	0.037
HAMD scores^b^	8.76 ± 6.58	11.64 ± 8.18	14.75 ± 8.45	6.983	0.001	0.081	<0.001	0.135
RBDQ-HK scores^b^	20.76 ± 19.12	24.68 ± 16.42	31.71 ± 18.77	3.346	0.038	0.354	0.012	0.188
**Quality of life**
PDQ-39 scores^b^	14.94 ± 10.86	24.20 ± 17.42	29.79 ± 15.22	14.391	<0.001	0.002	<0.001	0.137

*PD, Parkinson's disease; MHs, minor hallucinations; UPDRS, Unified Parkinson's disease rating scale; FAB, frontal assessment battery; MMSE, mini-mental state examination; PDSS, Parkinson's disease sleep scale; RBDQ-HK, rapid eye movement sleep behavior disorder questionnaire Hong Kong; HAMD, Hamilton depression scale; PDQ-39, 39-item Parkinson's disease questionnaire; LEDD, levodopa equivalent daily dose*.

a *Data were performed for group differences with the chi-squared test*.

b *Data were performed for group differences with ANOVA. P1, post-hoc comparisons p-value of isolated MHs group vs. PD-control group; P2, post-hoc comparisons p-value of MHs plus group vs. PD-control group; P3, post-hoc comparisons p-value of isolated MHs group vs. MHs plus group*.

In the demographic features, including sex, age, onset age, disease duration, and LEDD, there were statistical differences in age, disease duration, and LEDD among these three groups (*p* < 0.05). The age of patients in the complex MH group (71.42 ± 7.19 years) was older than that in the PD-control group (67.13 ± 7.54 years, *p* = 0.013), while it was similar with the isolated MH group (69.32 ± 8.45 years). The highest LEDD among the three groups was in the complex MH group (654.69 ± 337.35), which was significantly higher than that in the PD-control group (451.28 ± 267.72, *p* = 0.002) and that in the isolated MH group (465.25 ± 234.95, *p* = 0.017). However, no difference was found between the PD-control and isolated MH groups (*p* = 0.823). Disease duration was longer in the isolated MH group than in the PD-control group (8.80 ± 4.94 years vs. 6.84 ± 3.60 years, *p* = 0.037). There was no statistical difference in sex and onset age among these three groups.

Among the three groups, patients who had the most severe motor symptoms based on UPDRS-III scores were in the complex MH group (29.33 ± 11.32), followed by those in the isolated MH group (23.80 ± 13.25). The UPDRS-III scores of these two groups were both higher than that in the PD-control group (18.81 ± 8.71), (*p* < 0.001 and 0.031, respectively). Similarly, patients in the complex MH and isolated MH groups showed more advanced H-Y stages than the PD-control group. However, these differences were not found between the isolated MH and complex MH groups. In addition, there were great differences among the three groups with respect to NMSs, including depressive symptoms (*F* = 6.983, *p* = 0.001), sleep (*F* = 6.924, *p* = 0.001), memory (*F* = 6.004, *p* = 0.004), frontal symptoms (*F* = 15.399, *p* < 0.001), and REM Sleep Behavior Disorder (RBD) (*F* = 3.346, *p* = 0.038). Compared with the PD-control group, the complex MH group had more serious NMSs with higher HAMD scores (14.75 ± 8.45 vs. 8.76 ± 6.58, *p* < 0.001), higher Rapid Eye Movement Sleep Behavior Disorder Questionnaire scores (31.71 ± 18.77 vs. 20.76 ± 19.12, *p* = 0.012), lower PDSS scores (104.58 ± 23.31 vs. 120.13 ± 17.15, *p* < 0.001), lower FAB scores (14.75 ± 3.42 vs. 17.22 ± 1.33, *p* < 0.001), and lower MMSE scores (26.08 ± 2.92 vs. 27.78 ± 2.20, *p* = 0.005). While in the isolated MH group, only FAB and MMSE scores were lower than those in the PD-control group. However, these differences were not found between the isolated MH and complex MH groups, except for PDSS scores (lower in the MHs plus groups, *p* = 0.037).

Consequently, both the complex MH and isolated MH groups experienced poorer qualities of life with lower PDQ-39 scores (29.79 ± 15.22 and 24.20 ± 17.42, respectively), compared with the PD-control group (14.94 ± 10.86) (*p* < 0.001 and *p* = 0.002, respectively), while there was no statistical difference between the isolated MH and complex MH groups (*p* = 0.137).

### Association Between MHs and Clinical Variables

We analyzed the associated factors of MHs. The variables with significant differences between groups were then included in bivariable logistic regression (Table 2). As a dependent variable, the presence of MHs was defined as a binary variable. Age, disease duration, LEDD, UPDRS-III scores, H-Y stage, FAB scores, PDSS scores, HAMD scores, and MMSE scores were included as co-variates. Finally, advanced H-Y stage (OR: 2.697, 95% CI: 1.307–5.564, *p* = 0.007) and lower FAB score (OR: 0.684, 95% CI: 0.531–0.882, *p* = 0.003) were independent risk factors for MHs. In addition, logistic regression was conducted to differentiate between those with MHs and complex MHs with age, sex, LEDD, UPDRS-III scores, FAB scores, and PDSS scores as independent variables. After adjusting for age and sex, LEDD (OR: 1.003, 95% CI: 1.000–1.005, *p* = 0.031) were independent risk factors for complex MHs.

**Table 2:** Logistic regression analyses of the factors associated with minor hallucinations.

**Variables**	**PD-control group**	**PD with MHs group**	***t*/χ^2^-**	***p*-value**	**Multivariate**	
	**(*n* = 89)**	**(*n* = 49)**	**value**		
					**OR (95% CI)**	***p*-value**
Female sex, *n* (%)^a^	45 (50.56%)	21 (31.8%)	0.752	0.386	–	–
Age (years)^b^	67.13 ± 7.54	70.35 ± 7.13	−2.440	0.016	–	–
Onset age (years)^b^	60.31 ± 7.917	61.67 ± 8.53	−0.939	0.350	–	–
Disease duration (years)^b^	6.84 ± 3.60	8.67 ± 4.85	−2.520	0.013	–	–
LEED (mg/day)^b^	451.28 ± 267.72	558.04 ± 302.14	−2.141	0.034	–	–
UPDRS-III scores^b^	18.81 ± 8.71	26.51 ± 12.53	−3.824	<0.001	–	–
H-Y stage^b^	2.11 ± 0.55	2.62 ± 0.67	−4.880	<0.001	2.697 (1.307–5.564)	0.007
Dyskinesia, *n* (%)^a^	6 (6.74%)	9 (18.4%)	4.409	0.036	–	–
Wearing-Off, *n* (%)^a^	54 (60.67%)	29 (59.2%)	0.029	0.864	–	–
FAB scores^b^	17.22 ± 1.33	15.31 ± 2.97	4.272	<0.001	0.684 (0.531–0.882)	0.003
MMSE scores^b^	27.78 ± 2.20	26.20 ± 3.10	3.143	0.002	–	–
PDSS scores^b^	120.13 ± 17.15	110.18 ± 20.66	3.030	0.003	–	–
HAMD scores^b^	8.76 ± 6.58	13.16 ± 8.37	−3.177	0.002	–	–
RBDQ-HK scores^b^	20.76 ± 19.12	28.12 ± 17.78	−2.217	0.028	–	–
PDQ-39 scores ^b^	14.94 ± 10.86	26.94 ± 16.45	−4.579	<0.001	–	–

*PD, Parkinson's disease; MHs, minor hallucinations; UPDRS, Unified Parkinson's disease rating scale; FAB, frontal assessment battery; MMSE, mini-mental state examination; PDSS, Parkinson's disease sleep scale; RBDQ-HK, rapid eye movement sleep behavior disorder questionnaire Hong Kong; HAMD, Hamilton depression scale; PDQ-39, 39-item Parkinson's disease questionnaire; LEDD, levodopa equivalent daily dose*.

a *Data were performed for group differences with the chi-squared test*.

b *Data were performed for group differences with t-test. A logistic regression analysis based on the forward stepwise method was conducted to determine the most significant variables which were independently correlated with MHs in PD*.

**Figure 2:**
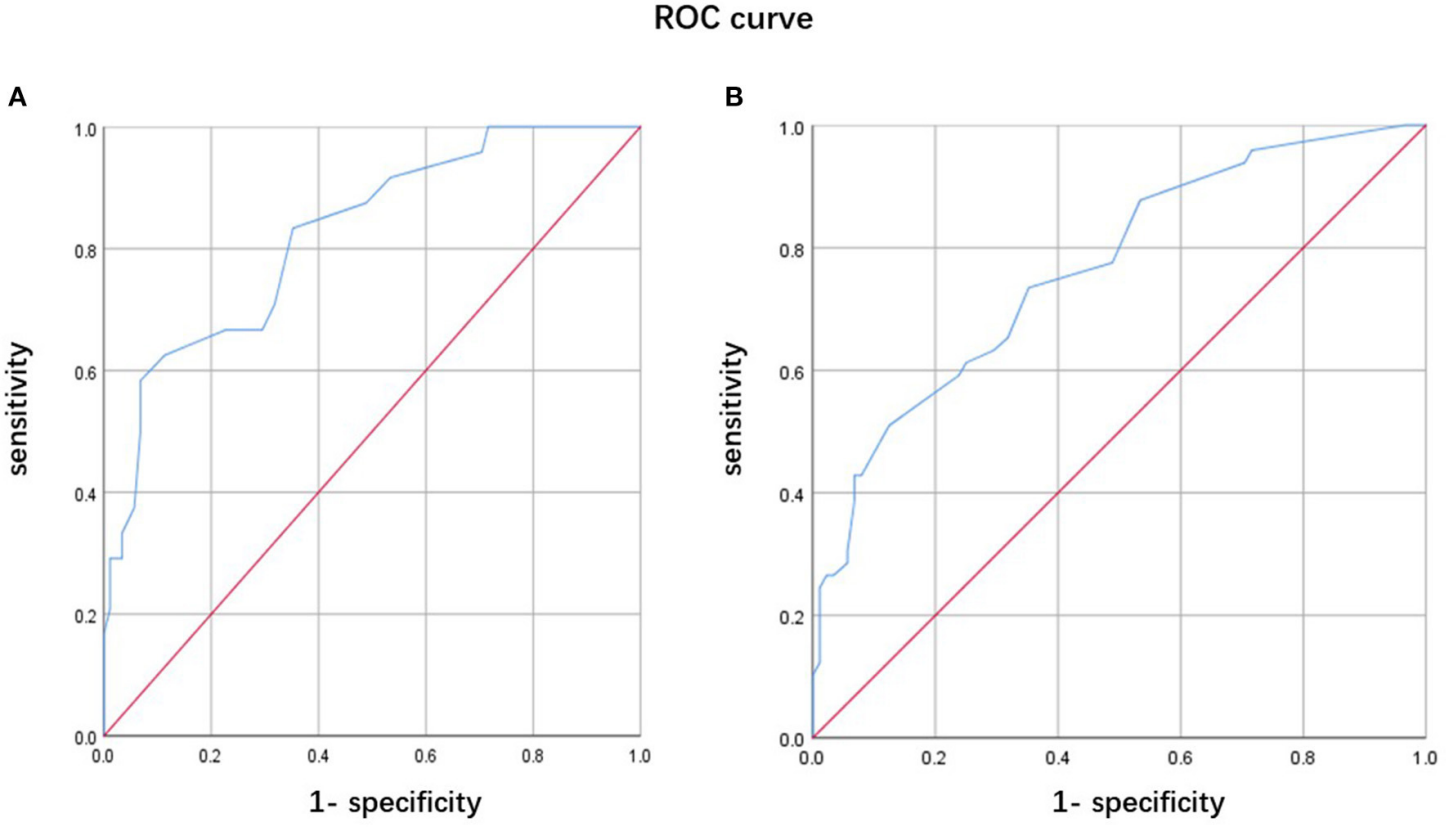
Receiver operating characteristic curve of predicting psychotic symptoms in Parkinson's disease (PD) patients. **(A)** A combination of the Hoehn-Yahr (H-Y) stage and FAB score for predicting complex minor hallucination (MH) occurrence in PD patients. **(B)** A combination of the H-Y stage and FAB score for predicting the MH occurrence in PD patients.

Because nearly half of the patients with MHs having well-structured hallucinations or delusions had tendencies of more severe motor, frontal, and sleep symptoms than patients with isolated MHs, we further analyzed predictors of complex MHs in PD, and its predictive values. Similarly, the risk factors were advanced H-Y stage (OR: 3.927, 95% CI: 1.468–10.503, *p* = 0.006) and lower FAB score (OR: 0.661, 95% CI: 0.490–0.891, *p* = 0.007). A ROC curve for predicting complex MHs occurrence in PD patients is shown in Figure 2A. A combination of the H-Y stage and FAB score was used to plot the ROC curve. The AUC was 0.822 (95% CI: 0.728–0.916). The sensitivity was 58.3% and the specificity was 93.2%. A ROC curve for predicting the occurrences of MHs in PD patients is shown in Figure 2B. A combination of the H-Y stage and FAB score was used to plot the ROC curve. The AUC was 0.765 (95% CI: 0.682–0.847). The sensitivity was 51.0% and the specificity was 87.5%.

## Discussion

Our results showed that the most common psychiatric symptoms in non-demented PD patients were minor hallucinations, and nearly half of them were isolated MHs. In general, PD patients with MHs (either isolated or complex) were worse than those without MHs, both in clinical profiles and quality of life. Advanced disease progression and more serious frontal symptoms were risk factors for the occurrence of MHs in PD patients, especially for predicting the presence of complex MHs. This result confirmed that the presence of minor hallucinatory phenomena in PD patients had important clinical and prognostic implications, which needed early prediction.

MHs were present in 32.9% of the PD patients, which was within the range reported in previous studies (16.9–44.8%) (Fenelon et al., 2000, 2010; Pacchetti et al., 2005; Mack et al., 2012; Frei and Truong, 2017; Omoto et al., 2021). Among them, isolated and complex MHs accounted for similar percentages (nearly 16%), which is consistent with other studies (Ffytche et al., 2017; Kulick et al., 2018). Although different investigators used different diagnostic tools, it was clear that the presence of MHs in PD patients was much higher than that in age-matched control subjects (Fenelon et al., 2000), and MHs appeared as a frequent and significant NMS. The phenomenology of MHs described in our study was similar to those previously described (Fenelon et al., 2000; Frei and Truong, 2017; Lenka et al., 2019). The passage hallucinations were the most common MHs, followed by illusions, followed by presence hallucinations. Our results showed that PD patients who had more than one type of MH all experienced presence hallucinations, although presence hallucinations were not the most common. Fenelon et al. also reported the importance of presence hallucinations in PD patients and showed that presence hallucinations were one of the predictors for well-structured hallucinations (Fenelon et al., 2011). This phenomenon suggested more careful screening for psychotic symptoms when a PD patient described presence hallucinations. Our results showed that the frequency of MHs in the middle-advanced PD stage was significantly higher than that in early PD patients, especially in patients in the complex MHs group. This indicated that the presence of MHs tended to progress in frequency with disease progression and that MHs could be observed, starting from the early stages of the disease. We also observed that MH occurred in earlier PD, while hallucinations occurred in more advanced stages of PD. However, this was an observational cross-sectional study, so recall bias could not be ignored. We plan to study the chronology of any form of hallucination and other motor or non-motor symptoms in a future follow-up study.

A prospective, longitudinal study has previously confirmed that MHs could predate the onset of Parkinsonism (Pagonabarraga et al., 2016). Furthermore, in our PD patients, the presence of MHs, mainly of complex MHs, were significantly associated with older age, advanced disease stages, and serious motor and non-motor symptoms, including frontal dysfunction, global cognitive impairment, depression, RBD, and sleep disorders. Regression analysis identified two factors that were independent predictors of MHs: severe frontal symptoms and advanced H-Y stages, especially for patients with complex MHs. This was consistent with the results of other studies (Lenka et al., 2019; Omoto et al., 2021). It was logical that as the disease progressed, the motor symptoms were more severe and more NMS appeared, including hallucinatory phenomena. A longitudinal study with a 2-year follow-up focused on PDP, indicated longer disease duration, worse disease severity, declines in cognitive performances, and the presence of depressive symptoms were associated with psychotic development (Morgante et al., 2012). Moreover, our finding showed that frontal symptoms were more present in patients with MHs, especially in complex MHs. The lower FAB scores might be a predictive factor for the presence of MHs, which was not mentioned in other studies. Some evidence indicated that executive dysfunction and inhibitory ability disturbance were risks for the development of hallucinatory experiences (Barnes and Boubert, 2008; Llebaria et al., 2010), mainly for well-formed visual hallucinations. One study showed that PD patients with MHs shared similar structural and functional brain abnormalities with patients with visual hallucinations (Bejr-Kasem et al., 2019). This suggested that if the frontal dysfunction was involved in the generation of visual hallucinations, it may be the same for MHs. However, another neuroimaging study reported decreased frontal lobe gray matter volume and glucose metabolism in PD patients with MHs, but this was associated with frontal cortex-mediated cognitive function rather than MHs (Ffytche et al., 2017). A recent study did not find atrophy of frontal areas in patients with MHs (Bejr-Kasem et al., 2021). Thus, frontal performance, whether as the main neural disturbance, contributing to the genesis of MHs, is uncertain. Long-term observation is therefore required to verify a causal relationship between MHs and frontal dysfunction.

Isolated MHs were slight and usually overlooked. However, in our cohort, the clinical profiles and qualities of life of patients with isolated MHs were more severe than those of the PD-control group, and closely resembled those of patients with complex MHs. The volumetric MRI combined with the FDG PET study also found no difference of neuroimaging parameters between patients with isolated MHs and MHs plus visual hallucinations (Ffytche et al., 2017), suggesting that the presence of even isolated MHs was clinically important.

In the past, hallucinations of PD were regarded as side effects of anti-Parkinsonian therapy, but this view has been controversial in recent years (Fenelon et al., 2000; Frei and Truong, 2017; Lenka et al., 2019). Evidence has indicated that hallucinations were part of PD, and may occur independently of medication (Pagonabarraga et al., 2016). Our data showed that patients with complex MHs received higher dosages of drug treatments, while no difference was found between the PD-control and isolated MH patients. Our PD patients with complex MHs tended to have higher motor symptom scores (*p* = 0.058), which led to augmented dosages of anti-PD drugs. In addition, the ratio of dopamine receptor agonists used did not differ among the three groups, so we did not find a clear association between MHs and anti-Parkinsonian drugs, suggesting that MHs resulted from the disease itself and might be a non-specific effect of the medications (Fenelon et al., 2000).

Our study had some limitations. First, our study was a cross-sectional study and did not include premorbid or *de novo* PD patients. Therefore, the causality between MHs and clinical variables could not be confirmed. It is, therefore, crucial to perform prospective studies to investigate causality. Second, all patients were recruited from a single movement disorder center. Finally, there was a small sample size and the questionnaires were limited in scope.

## Conclusion

More than one-third of PD patients in our cohort had MHs. Of these, half were isolated and half were complex. PD patients with MHs, even isolated MHs, had more severe motor and non-motor profiles than PD without MHs. The quality of life of the isolated MHs group more closely resembled that of the complex MH group, which was significantly decreased. Our results highlight the important clinical significance of MHs in PD patients, although this symptom has been considered “minor.” The early screening of MHs could be important, and the advanced stages and frontal dysfunctions might be predictors of MHs occurrence.

## Data Availability Statement

The original contributions presented in the study are included in the article/Supplementary Material, further inquiries can be directed to the corresponding author/s.

## Ethics Statement

The studies involving human participants were reviewed and approved by the Ethics Committee of XinHua Hospital affiliated to Shanghai Jiao Tong University School of Medicine. The patients/participants provided their written informed consent to participate in this study. Written informed consent was obtained from the individual(s) for the publication of any potentially identifiable images or data included in this article.

## Author Contributions

Analysis and interpretation of data and drafting and critical revision of the manuscript by YZ and GZ. Enrollment of patients and critical revision of the manuscript by XZ and ZZ. Critical revision of the manuscript and study supervision by JG and ZL. All authors contributed to the article and approved the submitted version.

## Funding

This study was supported by the National Key R&D Program of China (2017YFC1310300), Shanghai Health and Family Planning Commission Foundation (201940021), and Shanghai Pujiang Program (2020PJD032).

## Conflict of Interest

The authors declare that the research was conducted in the absence of any commercial or financial relationships that could be construed as a potential conflict of interest.

## Publisher's Note

All claims expressed in this article are solely those of the authors and do not necessarily represent those of their affiliated organizations, or those of the publisher, the editors and the reviewers. Any product that may be evaluated in this article, or claim that may be made by its manufacturer, is not guaranteed or endorsed by the publisher.
